# From Innate Spatial Biases to Enculturated Spatial Cognition: The Case of Spatial Associations in Number and Other Sequences

**DOI:** 10.3389/fpsyg.2018.00415

**Published:** 2018-03-29

**Authors:** Koleen McCrink, Maria Dolores de Hevia

**Affiliations:** ^1^Department of Psychology, Barnard College, Columbia University, New York, NY, United States; ^2^Université Paris Descartes, Paris, France; ^3^Laboratoire Psychologie de la Perception, CNRS UMR 8242, Paris, France

**Keywords:** space, number, neonates, laterality, toddlerhood

Humans, as well as other animals, use space to organize the world. This use of space as an organizational scaffold is especially prevalent when we conceptualize mathematics, a domain that shares behavioral and neural overlap with the domain of space (Pinel et al., 2004; Kaufmann et al., 2005; Dehaene and Brannon, 2010). One of the most prominent descriptions of this relation is that of a mental number line, in which small values are associated with the left side of space, and large values with the right (Moyer and Landauer, 1967; Dehaene et al., 1993). The development of the mature form of this mental number line is multiply determined, with evidence pointing to evolutionary pressures as well as cultural and linguistic influences. This cognitive bias to associate numerical information with space, and do so with left-right or right-left asymmetry, is adaptive; it helps to bolster memory and learning throughout our lives (Opfer and Furlong, 2011; McCrink and Galamba, 2015; McCrink and Shaki, 2016; Bulf et al., 2017). Moreover, with development this bias to map number onto an oriented continuum extends to any well-ordered information, even when recently learned (Gevers et al., 2003, 2004; Previtali et al., 2010). Critically, despite the apparent promise of using space as a scaffold for learning and memory, there are several gaps in the literature surrounding an essential period of the development of spatial-numerical associations: toddlerhood and early childhood. Here, we summarize current work on the innate and culture-specific factors modulating the mental number line in infancy and childhood, and note further research that could help to shed light on a complete developmental picture of this phenomenon.

## The mental number line: from innate to enculturated

Recent work in developmental psychology has found that spatial-numerical associations are present as early as the first days of life. de Hevia and colleagues have documented a propensity for infants in the first year of life to map magnitudes onto a left-to-right spatial continuum. Seven-month old infants present a preference for increasing numerical sequences, only if the arrays are presented from smallest on the left to largest on the right (de Hevia et al., 2014). Eight-month-olds are quicker to attend to a left-side probe after central presentation of a small number and a right-side probe after central presentation of a large number, but this advantage does not extend to a small vs. large object (Bulf et al., 2016). Interestingly, despite numerical magnitude and spatial quantity sharing many commonalities in infancy [e.g., an advantage for increasing order (Macchi Cassia et al., 2012; de Hevia et al., 2014, 2017a), transfer of ordinal direction and rule-based learning between the two domains (de Hevia and Spelke, 2010; Lourenco and Longo, 2010)], the findings of lateralized asymmetry for attention in infancy seem to be specific to numerical magnitude (e.g., sets of objects) and not spatial quantity (e.g., the size of a single object; Bulf et al., 2016; de Hevia et al., 2017b). This lateralized processing can be found even when the dimension evokes number only peripherally, such as when processing a statistical ordering rule for the placement of three objects (Bulf et al., 2017). The biases observed in infancy are untrained and spontaneous, reflecting predispositions for lateralized processing of magnitude. However, it is possible that by several months of age, infants have had some non-specific spatial experience that could lead to enculturation of a spatial organization system. de Hevia et al. (2017a) have recently found that even neonates exhibit lateralized processing of magnitude; they look longer to a left-side stimulus in the presence of a relatively small magnitude, and longer to a right-side stimulus in the presence of a relatively large magnitude. This finding—which is not mutually exclusive with a later, enculturated mental number line—supports the existence of a mental number line in humans with no prior spatial experience.

**Figure 1:**
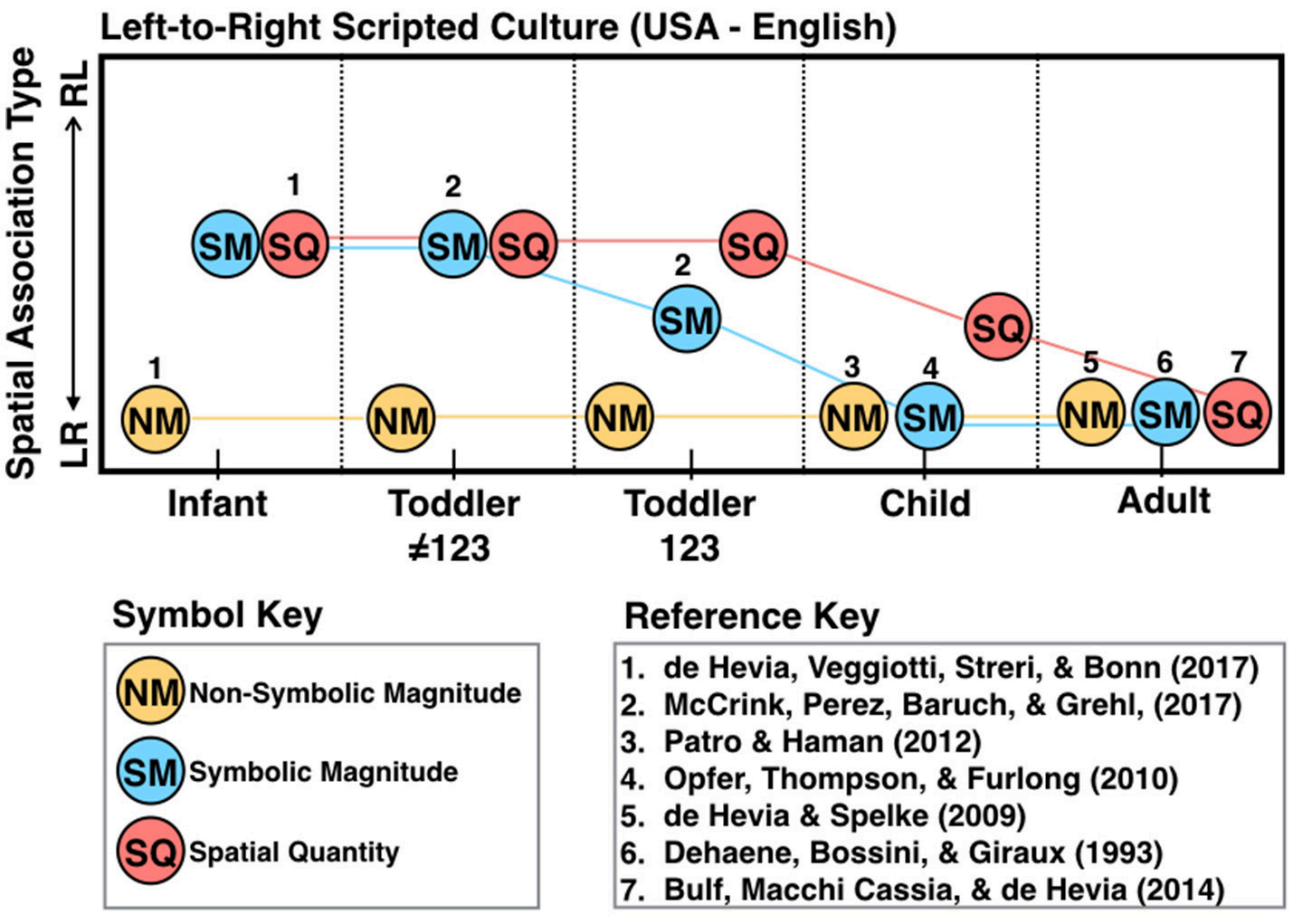
A summary of likely trajectories of the spatial association types [ranging from left-small/less and right-large /more (LR) to right-small/less and left-large /more (RL)] in early childhood, for a child whose language is consistently scripted left to right. Knowledge of the count list is indicated here by “123.” Numbers below the symbols indicate the studies which have been done to establish this trajectory, with author details noted on the reference key. A lack of numbers indicates an area for future work. In infancy, children spontaneously associate small and large magnitudes with the left and right sides of space (respectively). In the toddler years, children develop symbolic knowledge of the order of numerals, and are enculturated to the different spatial structures within their script for these symbols, which eventually prompts culture-specific spatial associations for many types of ordered sequences.

McCrink et al. (2017b) posited that these lateralized spatial-numerical associations wax and wane throughout infancy and early childhood as children become less beholden to innate biases, and more imitative and aware of the cultural conventions surrounding spatial structuring. In this study, 2- and 3-year-olds were given a version of a navigational spatial transposition task frequently used with non-human animals (Rugani et al., 2010; Drucker and Brannon, 2014). In the experimental conditions relevant to this review, toddlers were trained to retrieve an object that was repeatedly hidden in one particular location (out of 5) along a vertical array, with the experimenter verbally labeling the locations with numerals (“box one”) or a non-ordinal label (“this box”). Afterwards, the array was surreptitiously transposed 90 degrees. Unlike non-human animals, who exhibit a general bias to search from left-to-right after being trained in this spatially ordered sequence of locations, the children who received generic labels were equally likely to navigate with a LR or RL bias. However, children who received numerical labels selected the location that corresponded to a left-to-right spatial mapping. Moreover, in a counting task only ~60% of toddlers counted in an organized direction, and those were the children who reliably performed a left-to-right mapping. In light of these findings, the authors suggest that toddlerhood is a period of flexibility with respect to the directional nature of spatial associations, with innate left-to-right scanning biases falling away as children begin to gather socially transmitted information of the spatial structuring in their environment. Early biases to map initial information to the left side of space, and final to the right, will arise only if the privileged domain of number is invoked (See Figure 1 for the proposed developmental trajectory of several types of spatial associations).

This privileged mapping of numerals to space is likely due to the combination of the children's knowledge of the mapping between numerals and magnitude (an inherently ordinal dimension), and the reinforcement of left-to-right spatial structuring by their caregivers when counting. During the preschool years, children start to reliably map small numbers (“1, 2, 3”) to their innate, non-symbolic, and intrinsically ordered representations of number (Sarnecka and Carey, 2008). By preschool, children show spatial-numerical compatibility effects similar to older children and adults for non-symbolic magnitudes (de Hevia and Spelke, 2009; Patro and Haman, 2012), and are more likely to use symbolic numerical labels to solve a spatial reasoning task if they are presented in a culturally consistent direction (Opfer et al., 2010). In this paradigm (adapted from Loewenstein and Gentner, 2005), preschoolers are shown two sets of boxes (a sample and matching set), sectioned into verbally labeled locations (e.g., “room 2”). A target is shown in the sample set, and children search for this target in the matching set (located in the same labeled location). Preschoolers in the U.S. are faster and more accurate when locations are numbered from left-to-right versus right-to-left, if they are highly organized counters (Opfer et al., 2010). Additionally, Shaki et al. (2012) found that preschoolers in cultures with right-to-left scripted language (such as Arabic) exhibit spatial-numerical biases that are reversed, with young children counting from right-to-left instead of from left-to-right as they do in English-speaking countries.

How may this conventionality emerge? Given the timing of this shift, the obvious candidate is the child's home environment. Starting in early toddlerhood, caregivers are modeling the spatial conventions of their culture, presenting spatial associations with a high degree of culture-specific structure. Parents may primarily model a single effective strategy when they organize space for their child—a strategy that is colored by the language they read and write on a daily basis. Recent work on caregiving influences on spatial biases suggests there are three primary ways that parents can influence their child's spatial structuring habits: their gesture, their organization of spatial layout, and the nature of their reading material (Patro et al., 2016a; Göbel et al., 2017; McCrink et al., 2017a). McCrink et al. (2017a) found that in two different tasks—watching a slideshow of alphabetical, numerical, or random stimuli, and crafting a visual story for their child –English-speaking parents were more likely to gesture to the screen and lay out pictures in a left-to-right manner to a greater degree than Hebrew-speaking parents. Göbel et al. (2017) found that after observing reading from storybooks (a left-to-right or right-to-left storybook) children change their counting direction in line with the direction of reading. Observing an adult point in a specific direction (e.g., right to left) did not influence counting direction. In contrast, Patro et al. (2016b) found that if the children were trained by an adult to point in a specific direction themselves, their subsequent spatial-numerical mappings took on the asymmetric form of that pointing movement (left-less/right-more after left-to-right pointing, and right-less/left-more after right-to-left pointing). Finally, book illustrations exhibit culture-specific directionality, even in non-numerical domains, with the subject[object] of the sentence on the left[right] for English-language books, and the opposite for Hebrew-language books (Göbel et al., 2017). The accumulation of this cultural experience results in an asymmetric mapping for many types of ordinal information (numerical: Dehaene et al., 1993; Zebian, 2005, spatial quantity: Bulf et al., 2014, alphabetical: McCrink and Shaki, 2016)—a mapping which follows the direction of the culture's script.

## Future directions on the early development of the mental number line

Several outstanding questions remain within this subfield. First, is the number-space mapping in infancy actually related to the ubiquitous spatial associations found in adulthood? It is instead possible that these are two separate phenomena, which reflect different underlying mechanisms [e.g., hemispheric lateralization influences in infancy, but a distinct symbolic, analogical reasoning system starting in the second year of life Halford et al., 2010, 2013]. One way to address this possibility is to investigate both the structure and function of brain areas which respond to numerical and spatial magnitudes (e.g., Borghesani et al., 2016), and observe if there is continuity across development with respect to which regions are activated in similar tasks. Second, what is the underlying spatial relation between different types of quantity representations at birth? Studies which investigate the numerical specificity of spatial associations in neonates should be conducted in order to detail how the domain of number is structured and reasoned about. Third, when does the enculturation shift for spatial associations happen—and does the presence or absence of numerical input alter this timeline? To answer this question, research is needed in which the same spatial association task is implemented in infants, toddlers, and children in cultures which observe left-to-right and right-to-left scripting behaviors. One good candidate would be the spatial transposition task, which requires no verbal knowledge, and can be altered for the presence or absence of non-symbolic number arrays on each location. Fourth, how exactly is this enculturation of spatial associations implemented? Work on spatial enculturation behaviors like gesturing along a path (Patro et al., 2016b) and reading (Göbel et al., 2017) has started to document possible avenues, but a closer study of the home environment and the relation between parent behaviors and child spatial associations is needed. For example, if reading observation is a primary avenue to enculturation for this phenomenon, one would predict that highly literate homes would have children who exhibit a quicker and more robust transition to the spatial associations of their culture. Additionally, a causal story for parent interaction as the driver of enculturated spatial associations would predict that parents' degree of spatial structuring would be the modulating factor in their child's degree of spatial associations. Finally, the relation between different types of enculturation behaviors and different types of numerical representations is still unclear. Developmental studies which systematically tease apart the influence of these behaviors (a parent modeling spatial organization vs. a child mimicking these modeled behaviors, parental modeling of spatial organization in a numerical or non-numerical fashion) and representations (explicit counting, non-symbolic mapping of magnitudes) could help clarify the nature of the mental number line in early childhood.

## Author contributions

KM and MDdH contributed equally to the generation of this opinion. KM drafted the manuscript. MDdH provided comments.

### Conflict of interest statement

The authors declare that the research was conducted in the absence of any commercial or financial relationships that could be construed as a potential conflict of interest.
